# Treatment of Acquired von Willebrand Syndrome and Prevention of Bleeding Postautologous Stem Cell Transplant during Severe Pancytopenia with IVIG

**DOI:** 10.1155/2015/809313

**Published:** 2015-04-02

**Authors:** Behyar Zoghi, Paul Shaughnessy, Roger M. Lyons, Richard Helmer, Carlos Bachier, C. Frederick LeMaistre

**Affiliations:** ^1^Adult Blood and Marrow Transplant, Texas Transplant Institute, San Antonio, TX, USA; ^2^Sarah Cannon Blood Cancer Network, Nashville, TN 37203, USA; ^3^Cancer Care Centers of South Texas, 4411 Medical Drive, San Antonio, TX, USA; ^4^Texas Oncology, 901 W. 38th Street, Suite 200, Austin, TX 78705, USA

## Abstract

The use of high dose chemotherapy followed by autologous hematopoietic stem cell transplantation for remission consolidation after initial induction represents standard of care for patients with multiple myeloma. Patients with myeloma and Acquired von Willebrand Syndrome (AVWS) undergoing autologous stem cell transplant (ASCT) are at significant risk of bleeding due to the profound thrombocytopenia, low Factor VIII levels, fever, and toxicities associated with the preparative regimen. We report a patient with AVWS associated with multiple myeloma who underwent autologous stem cell transplants as consolidation after initial induction and again at relapse. He was successfully treated with high dose intravenous immunoglobulin (IVIG) prior to each transplant with rapid resolution of AVWS.

## 1. Introduction

The use of high dose chemotherapy followed by autologous hematopoietic stem cell transplantation (ASCT) for remission consolidation after initial induction therapy can increase disease-free and overall survival in some patients with multiple myeloma [1]. Lymphoproliferative disorders and multiple myeloma appear to be most frequently associated with AVWS accounting for 48–63% of cases [2]. AVWS usually resolves when the underlying associated condition disappears.

The Acquired von Willebrand Syndrome (AVWS) is a rare acquired bleeding disorder in which von Willebrand factor (VWF) is synthesized normally but immune complexes form with nonspecific antibodies associated with the primary disease leading to enhanced clearance by the reticuloendothelial system. AVWS is typically characterized by low VWF and Factor VIII levels and no past or family history of bleeding.

Patients with myeloma and AVWS undergoing ASCT are at significant risk of bleeding due to the profound thrombocytopenia, low Factor VIII levels, fever, and toxicities associated with the preparative regimen. There is little literature guiding the most effective therapies to control bleeding with AVWS during the pancytopenic phase after ASCT. We report a patient with AVWS associated with multiple myeloma that received two autologous stem cell transplants separated by two years. In each instance, the patient was treated with high dose intravenous immunoglobulin (IVIG) with a rapid resolution of AVWS within a few days with a persistent response lasting until day 100 after ASCT.

## 2. Case Presentation

In 2008, a 66-year-old insulin dependent patient taking daily aspirin therapy with no past or family history of bleeding was visiting family in another city. He presented to the local emergency room with spontaneous rectal bleeding. He received packed red blood cells, fresh frozen plasma, and cryoprecipitate and underwent a partial colon resection with resolution of bleeding. He was tentatively diagnosed with von Willebrand disease (with ristocetin cofactor activity <20%, Factor VIII 7–16%, and VWF-Ag 14% with normal multimers in 2008), discharged from hospital, and returned home where he was then followed without further bleeding. Even though the multiple myeloma was diagnosed in 2011, he may have had smoldering myeloma and might have had an early manifestation of slow progression of myeloma. He received desmopressin (DDAVP) and Amicar for dental resections and later for cystoscopy and removal of bladder stones. He had no bleeding with either procedure until fourteen days after the cystoscopy when he developed hematuria and urinary retention requiring hospitalization and Factor VIII replacement. He was later diagnosed with symptomatic coronary artery disease and he underwent coronary artery bypass grafting (12/16/2010) with Factor VIII support. During surgery, however, the sternum was found to be thin and fragile and a biopsy showed cells diagnostic of a plasmacytoma. A bone marrow biopsy showed 10% plasma cells with normal cytogenetics and a skeletal survey showed multiple lytic lesions throughout axial and appendicular skeleton. Renal function and calcium were normal, total protein was 6.3, SPEP showed 0.5 gm/dL M-spike, and albumin was 1.9.

Following recovery from surgery, he received four cycles of lenalidomide, bortezomib, and dexamethasone and achieved a partial remission. He was referred for stem cell transplant and underwent mobilization with filgrastim and plerixafor. Factor VIII concentrate was given prior to insertion of Quinton catheter, but he experienced local bleeding despite the simultaneous use of Amicar. The bleeding at the catheter site was minimal and the patient was able to proceed with collection. A total of 5.12 × 10^6^ CD-34 cells/kg were collected in three apheresis procedures. The preparative regimen consisted of melphalan at 140 mg/m^2^ followed by 3.1 × 10^6^ cryopreserved CD-34 cells/kg for the first transplant.

Given the data regarding the role of high dose IVIG in treatment of VWF disease [3] and the bleeding associated with Quinton catheter placement, the patient was treated with high dose IVIG given at 1 gm/kg daily on days −2 and −1 [4, 5]. Von Willebrand factor antigen was 13% before IVIG was given. Six days after the first dose, The VWF was 236% and after eleven days it was 251%. No inhibitor of Factor VIII level was detected (Table 1). His inpatient stay was complicated by the expected stomatitis, nausea, and diarrhea, all of which were grade 2 or less. He received filgrastim 480 *μ*g subcutaneously from day +7 to day +12. His only transfusion requirements were platelet transfusions on days +8 and +10 and two units of red blood cell transfusions on day +10. He reached >500/*μ*L absolute neutrophils and >20,000/*μ*L platelets on day +12 after transplant, and there were no bleeding episodes during hospitalization, except minor gingival oozing. By day 100, the patient's light chains had normalized, but a small monoclonal protein of 0.13 gm/dL was detectable.

**Figure 1:**
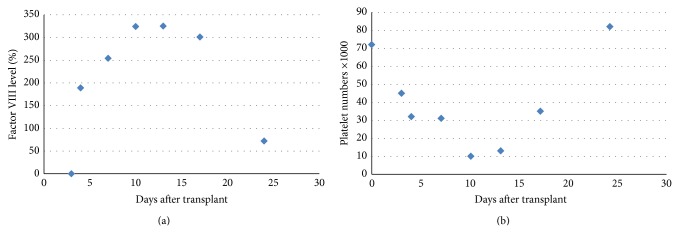
(a) Factor VIII level started to rise 4 days after transplant and slowly tapered off and stayed elevated up to 100 days after transplant. (b) Despite the fact that patient had severe thrombocytopenia by day +10 after transplant, no bleeding episodes were noted. (a) At the same time, the level of Factor VIII was the highest level which provided the protective role on bleeding prevention.

He remained on maintenance Revlimid and dexamethasone until hospitalized in January 2013 following a syncopal episode. He was found to have a high grade AV nodal block requiring pacemaker insertion. During the hospitalization, a bone marrow biopsy showed 20–25% plasma cell with translocation (11:14). The skeletal survey showed evidence of progressive disease. He underwent two cycles of carfilzomib and achieved VGPR followed by the second high dose chemotherapy and autologous stem cell transplant. Two months previously, Factor VIII level was 12% and on the day of admission the level of Factor VIII was undetectable. IVIG treatment again increased Factor VIII levels significantly over the following days (Figure 1(a)). Despite significant thrombocytopenia at the same time, no bleeding events occurred (Figure 1(b)). The transplant course was complicated with neutropenic fever, toe infection, nausea, diarrhea, dehydration, and failure to thrive with no bleeding complication. Day +100 after transplant showed SPEP with no M spike, normal kappa and lambda light chain with IgG 487, IgA 27, and IgM 21, and normal SPE on day +100. The bone marrow examination (day 351) showed less than 5% plasma cells and Factor VIII level again became low (13%).

## 3. Discussion

AVWS has been associated with various underlying hematological diseases, most commonly lymphoproliferative disease: monoclonal gammopathies and myeloproliferative disorders [3]. Three pathogenic mechanisms appear to govern the increased plasma clearance of VWF: (1) adsorption of VFW onto malignant cells or platelet, (2) increase of proteolysis due to high shear stress such as that in aortic stenosis, and (3) binding of VFW by antibodies that form circulating complexes. The latter may result from either autoantibody to Factor VIII/VWF or nonspecific antibodies that form complexes cleared by Fc bearing cells. There are multiple options for the treatment of AVWF [6] including DDAVP, recombinant Factor VIIa and Factor VIII concentrate, Amicar, and IVIG. Responses to DDAVP are variable, ranging between 21% for myeloproliferative disorders, 44% for lymphoproliferative diseases and others, and 75% for other neoplastic diseases. Disadvantages of other therapies include a short half-life for VWF containing concentrate and a higher chance of thrombosis in older patients with heart disease for recombinant Factor VIIa. Amicar may be useful as adjuvant therapy in patients with thrombocytopenia. IVIG increases VWF rapidly and is associated with a prolonged response, up to 100 days as seen in our patient, making it an attractive agent for autologous stem cell transplant. Clinical response to IVIG and increase Factor VIII implies an effective therapy even though VWF antigen and cofactor were not collected. Despite the lack of antimyeloma therapy on the AVWS following the 2nd transplant when patient was in CR, there is a possibility that the patient might have minimal residual disease that is not detected which may be the reason why AWVS is still present despite the hematological response. Further follow-up can decipher this in future.

Our patient displayed the clinical and lab manifestations of AVWS even when he is in partial remission prior to transplant. He experienced further tumor reduction with high dose melphalan and ASCT twice, which may have extended the period in which he had normal Factor VIII levels beyond what might be predicted for IVIG therapy alone. In any case, IVIG appeared to be an effective approach in preventing bleeding associated with AVWFS during the periods of thrombocytopenia in two separate autologous stem cell transplants.

## Figures and Tables

**Table 1:** Factor VIII dilution studies.

	Day −2	Day 0	Day 11	Day 100
1:10	<6	135	179	66
1:20	<6	126	181	66
1:40	<6	118	193	60
1:80	<6	112		64
1:160	<6	120		72
1:320	<6	144		88
1:640	<6			384

There was no inhibitor of Factor VIII. Dilution did not show any changes in the % level in Factor VIII.

## References

[1] van Rhee F., Giralt S., Barlogie B. (2014). The future of autologous stem cell transplantation in myeloma. *Blood*.

[2] Federici A. B., Rand J. H., Bucciarelli P. (2000). Acquired von Willebrand syndrome: data from an international registry. *Thrombosis and Haemostasis*.

[3] Tiede A., Rand J. H., Budde U., Ganser A., Federici A. B. (2011). How I treat the acquired von Willebrand syndrome. *Blood*.

[4] van Genderen P. J. J., Terpstra W., Michiels J. J., Kapteijn L., van Vliet H. H. D. M. (1995). High-dose intravenous immunoglobulin delays clearance of von willebrand factor in acquired von Willebrand disease. *Thrombosis and Haemostasis*.

[5] Hanley D., Arkel Y. S., Lynch J., Kamiyama M. (1994). Acquired von Willebrand's syndrome in association with a lupus-like anticoagulant corrected by intravenous immunoglobulin. *American Journal of Hematology*.

[6] Federici A. B., Stabile F., Castaman G., Canciani M. T., Mannucci P. M. (1998). Treatment of acquired von Willebrand syndrome in patients with monoclonal gammopathy of uncertain significance: comparison of three different therapeutic approaches. *Blood*.

